# Analysis of Stevens-Johnson syndrome and toxic epidermal necrolysis using the Japanese Adverse Drug Event Report database

**DOI:** 10.1186/s40780-016-0048-5

**Published:** 2016-06-21

**Authors:** Junko Abe, Ryogo Umetsu, Kanako Mataki, Yamato Kato, Natsumi Ueda, Yoko Nakayama, Yuuki Hane, Toshinobu Matsui, Haruna Hatahira, Sayaka Sasaoka, Yumi Motooka, Hideaki Hara, Zenichiro Kato, Yasutomi Kinosada, Naoki Inagaki, Mitsuhiro Nakamura

**Affiliations:** Laboratory of Drug Informatics, Gifu Pharmaceutical University, 1-25-4 Daigaku-nishi, Gifu, 501-1196 Japan; Medical Database Co., Ltd, 3-11-10 Higashi, Shibuya-ku, Tokyo 150-0011 Japan; Molecular Pharmacology, Department of Biofunctional Evaluation, Gifu Pharmaceutical University, 1-25-4 Daigaku-nishi, Gifu, 501-1196 Japan; Department of Pediatrics, Gifu University Graduate School of Medicine, 1-1 Yanagido, Gifu, 501-1194 Japan; United Graduate School of Drug Discovery and Medical Information Sciences, Gifu University, 1-1 Yanagido, Gifu, 501-1194 Japan; Laboratory of Pharmacology, Gifu Pharmaceutical University, 1-25-4 Daigaku-nishi, Gifu, 501-1196 Japan; Clinical Research, Innovation and Education Center, Tohoku University Hospital, 1-1 Seiryo-machi, Aoba Ward, Sendai, Miyagi 980-8574 Japan

**Keywords:** Stevens-Johnson syndrome, Toxic epidermal necrolysis, Japanese Adverse Drug Event Report

## Abstract

**Background:**

Stevens-Johnson syndrome (SJS) and toxic epidermal necrolysis (TEN) are severe cutaneous adverse reactions associated with fatal disorders. Although many causes of SJS/TEN have been proposed, the time-to-onset for SJS/TEN and the relationship between aging and SJS/TEN are still not clear. Therefore, the aim of this study was to determine the relationship between aging and SJS/TEN using the Japanese Adverse Drug Event Report (JADER) database and analyze the time-to-onset profile of SJS/TEN.

**Methods:**

We analyzed reports of SJS/TEN recorded in the JADER database between 2004 and 2015 using an adjusted reporting odds ratio (ROR). We also used Weibull proportional hazards models for each drug to examine the expression patterns of SJS/TEN. We selected the drugs according to the number of the reports associated with SJS/TEN.

**Results:**

The JADER contained 330,686 reports from April 2004 to April 2015. The adjusted RORs for patients in the 0–19-, 20–39-, 60–79-, and ≥ 80-year-old groups from all data extracted from the JADER database were 1.33 (95 % confidence interval [CI], 1.21–1.45), 1.78 (95 % CI, 1.65–1.93), 0.71 (95 % CI, 0.66–0.75), and 0.72 (95 % CI, 0.66–0.79), respectively. The adjusted ROR tended to be higher in patients aged 0–19 years, particularly in patients using antipyretic analgesics, such as loxoprofen or acetaminophen. More than half of the cases of SJS/TEN onset following administration of loxoprofen and acetaminophen occurred within 4 days of the initiation of treatment. The median times-to-onset were 3 days for loxoprofen and 2 days for acetaminophen. The scale parameter α values of loxoprofen and acetaminophen were 9.44 and 6.17, respectively. The upper 95 % CIs of shape parameter β values for the drugs were all less than 1, with the exceptions of those for carbamazepine, ACE inhibitors, and corticosteroids.

**Conclusions:**

Our results suggested that monitoring of younger patients who frequently use antipyretic analgesics is important. These drugs should be used and monitored within the first 2–3 days of treatment in the Japanese population.

## Background

Stevens-Johnson syndrome (SJS) and toxic epidermal necrolysis (TEN) are severe mucocutaneous blistering disorders [1]. SJS and TEN have a significant impact on public health because of their associated high mortality rates (1–10 % for SJS and 20–40 % for TEN) [1–3]. Although the pathogenesis of SJS/TEN is not fully understood, these disorders are thought to be related to dysfunctions of the immune system. Moreover, the occurrence of SJS/TEN is usually associated with hypersensitivity to medications [4–6].

The incidences of SJS and TEN are estimated to range from 1 to 6 and 1 to 3 cases per million person-years, respectively [1, 2, 7, 8]. Since SJS and TEN are very rare diseases, evaluation of the relationship between suspected medication and SJS/TEN onset is difficult. Generally, spontaneous reporting systems (SRSs) are useful for the detection of new, rare, and severe adverse events. We have already analyzed reports of SJS/TEN using the United States (U. S.) Food and Drug Administration (FDA) Adverse Events Reporting System (FAERS) and have demonstrated a relatively high value for the signal (reporting odds ratio [ROR]) of SJS/TEN in patients ages 17 years and younger [9].

The genetic features of human leukocyte antigen in individuals may affect the incidence of SJS/TEN [10]. As such, regional differences in drug prescriptions should be taken into account. In the Japanese population, the incidence of SJS/TEN and the relationship between aging and SJS/TEN have still not been assessed. The regulatory authority of Japan, i.e., the Pharmaceuticals and Medical Devices Agency (PMDA), has released an SRS of the Japanese Adverse Drug Event Report (JADER) database. The JADER database is the best-known database in Japan and is one of the primary tools used for pharmacovigilance [11]. Indexes for signal detection, such as ROR, have been developed for use in the SRS to identify drug-associated adverse events by disproportionality analysis [12–14].

The European SCAR Study (EuroSCAR Study) is an international multicenter case–control study designed for surveillance of medication risks for SJS/TEN [6]. Time latency between the beginning of drug use and the onset of adverse events has been demonstrated in the EuroSCAR Study [6]. Accordingly, analysis of time-to-onset has recently been proposed as a method to detect signals for adverse events in the SRS. The rate of occurrence of adverse events after prescription is thought to depend on the causal mechanism and will often vary over time; in contrast, adverse events not associated with the drug will occur at a constant background rate. Therefore, varying rates of adverse events over time may indicate a drug-event relationship. The Weibull shape parameter (WSP) test is used for the statistical analysis of time-to-onset data and can describe the varying incidence of adverse events (i.e., changes in risk over time). Additionally, time-to-onset analysis with WSP has been used to evaluate hazard functions for detecting adverse events.

In this study, we evaluated the relationship between aging and SJS/TEN using RORs adjusted by logistic regression analyses in the JADER database and compared these results with previous results obtained from the FAERS database. We also applied time-to-onset analysis using WSP tests to the evaluation of SJS/TEN reports. To the best of our knowledge, this study is the first to evaluate time-to-onset data for SJS/TEN using the SRS.

## Methods

### Data sources

Data from the JADER database, which has been used to record data from April 2004 to April 2015, were obtained from the PMDA website (www.pmda.go.jp). The structure of the JADER database complies with the international safety reporting guidelines (ICH E2B). The database consists of four data Table 1) patient demographics information (demo), 2) drug information (drug), 3) adverse events (reac), and 4) primary illness (hist). The JADER database does not contain the code for identification of case reports (A1.11); therefore, we did not perform data cleaning. The “Drug” file (drug information) contains the role code assigned to each drug: suspected drug (“higiyaku” in Japanese), interacting drug (“sougosayou” in Japanese), and concomitant drug (“heiyouyaku” in Japanese). In this study, we analyzed records of suspected drugs.

**Table 1:** SJS/TEN in the Japanese Adverse Drug Event Report database (April 2004–April 2015)

	Gender	Total			Allopurinol			Loxoprofen			Acetaminophen			Carbamazepine			Lamotrigine		
		Reported cases		Reporting ratio (%)	Reported cases		Reporting ratio (%)	Reported cases		Reporting ratio (%)	Reported cases		Reporting ratio (%)	Reported cases		Reporting ratio (%)	Reported cases		Reporting ratio (%)
		SJS or TEN	Total		SJS or TEN	Total		SJS or TEN	Total		SJS or TEN	Total		SJS or TEN	Total		SJS or TEN	Total	
Total		6568	353988	1.86	544	2642	20.59	525	4714	11.14	498	1925	25.87	420	4517	9.30	403	2375	16.97
Stratified by age^a^																			
≤19 years	M	388	12982	2.99	1	9	11.11	14	69	20.29	87	325	26.77	35	345	10.14	3	76	3.95
	F	295	12088	2.44	0	7	0.00	12	80	15.00	83	262	31.68	17	265	6.42	17	144	11.81
20-39 years	M	470	13842	3.40	22	98	22.45	89	368	24.18	32	129	24.81	41	369	11.11	32	191	16.75
	F	791	20302	3.90	11	39	28.21	100	555	18.02	101	298	33.89	54	481	11.23	82	554	14.80
40-59 years	M	723	36749	1.97	80	454	17.62	60	606	9.90	33	155	21.29	40	630	6.35	60	318	18.87
	F	796	36347	2.19	39	126	30.95	90	631	14.26	58	205	28.29	49	495	9.90	76	331	22.96
60-79 years	M	1192	86098	1.38	154	902	17.07	88	991	8.88	45	218	20.64	77	761	10.12	25	170	14.71
	F	1099	71275	1.54	106	420	25.24	53	889	5.96	45	198	22.73	76	569	13.36	53	251	21.12
≥80 years	M	227	18393	1.23	35	218	16.06	6	169	3.55	3	39	7.69	5	121	4.13	10	27	37.04
	F	383	22610	1.69	89	266	33.46	9	300	3.00	8	66	12.12	10	153	6.54	6	39	15.38
Subtotal		6364	330686	1.92	537	2539	21.15	521	4658	11.19	495	1895	26.12	404	4189	9.64	364	2101	17.33

^a^Completed with age and gender

### Definition of adverse events

According to the terminology preferred by the Medical Dictionary for Regulatory Activities (MedDRA) version 17.1 [15], which were coded in “reac” table, we extracted case reports of SJS/TEN. We used the following three preferred terms (PT) to match SJS and TEN: SJS/PT10042033, oculomucocutaneous syndrome/PT10030081, and TEN/PT10044223. We excluded the reports that were uncompleted with age or sex information.

### Signal detection

The PMDA and Netherlands Pharmacovigilance Center use the ROR to detect the signal of adverse events from the SRS [16]. The ROR was calculated from two-by-two contingency tables of the presence or absence of a particular drug and a particular adverse event in the case reports. The ROR is the ratio of the odds of reporting adverse events versus all other events associated with the drug of interest compared with the reporting odds for all other drugs present in the database [16]. Use of the ROR allows for adjustment through logistic regression analysis and offers the advantage of controlling for covariates [17].

In this analysis, we selected the drugs based on the quantity of reports and refined the results with a dedicated correction to detect possible confounders present in the database [17]. We calculated adjusted RORs for control of covariates, using logistic regression analysis. The reports were stratified by age as follows: ≤19 years, 20–39 years, 40–59 years, 60–79 years, and ≥80 years. To construct the logistic model, gender, reporting year, and stratified age groups were coded. In this study, we defined the youngest group as less than 20 years old, unlike in our previous research, because age is recorded in 10-year intervals in the JADER database. The following logistic model was used for analysis:

$$ \mathrm{Log}\ \left(\mathrm{odds}\right) = {\upbeta}_0+{\upbeta}_1\mathrm{Y} + {\upbeta}_2\mathrm{G} + {\upbeta}_3\mathrm{A} $$

where Y is the reporting year, G is gender, and A is the stratified age group.

For signal detection, general qualitative judgments were used. The detection of a signal was dependent on the signal indices exceeding a predefined threshold. ROR values of less than 1 indicated no potential exposure-event associations, and estimates of more than 1 indicated potential exposure-event safety signals. The safety signals were considered significant when the ROR estimates and the lower limits of the corresponding 95 % confidence interval (CI) were greater than or equal to 1 [16].

The adjusted RORs were calculated using the 40–59-year-old group as a reference group. A likelihood ratio test was used to evaluate the effects of explanatory variables. A probability (*p*) value of 0.05 or less was considered statistically significant because the difference in −2 log likelihood followed a chi-square distribution with one degree of freedom.

### Time-to-onset analysis

Time-to-onset duration from the JADER database was calculated from the time of the patient’s first prescription to the occurrence of the adverse events. The median duration, quartiles, and WSPs were used to evaluate the time-to-onset data [18].

The scale parameter α of the Weibull distribution determines the scale of the distribution function. A larger scale value (α) stretches the distribution, while a smaller scale value shrinks the data distribution. The shape parameter β of the Weibull distribution determines the shape of the distribution function. A larger shape value gives a left-skewed curve, whereas a smaller shape values gives a right-skewed curve. In the analysis of the SRS, the shape parameter β of the Weibull distribution was used to indicate the hazard without a reference population as follows. When β was equal to 1, the hazard was estimated to be constant over time. If β was greater than 1 and the 95 % CI of β excluded the value 1, the hazard was considered to increase over time. Finally, if β was less than 1 and the 95 % CI of β excluded the value 1, the hazard was considered to decrease over time [19, 20].

Data analyses were performed using JMP, version 11.0 (SAS Institute Inc., Cary, NC, USA).

## Results

The JADER contained 353,988 reports from April 2004 to April 2015. After extracting reports having complete gender and age information, 330,686 reports remained; these reports were used for the analysis. The numbers of reports obtained for each PT were 3,653 for SJS, 841 for oculomucocutaneous syndrome, and 2,183 for TEN. The numbers of cases and reporting ratios for allopurinol, loxoprofen, acetaminophen, carbamazepine, and lamotrigine were 544 (20.6 %), 525 (11.1 %), 498 (25.9 %), 420 (9.3 %), and 403 (17.0 %), respectively. Data describing these top five most frequent drugs are summarized in Table 1. The number of reported cases for the 60–79-year-old group was 2,291; this was the highest value among all age-stratified groups.

The adjusted RORs and 95 % CIs for the whole data and subset data are summarized in Table 2. The adjusted RORs for patients in the 0–19-, 20–39-, 60–79-, and ≥ 80-year-old groups from all data extracted from the JADER database were 1.33 (95 % CI, 1.21–1.45), 1.78 (95 % CI, 1.65–1.93), 0.71 (95 % CI, 0.66–0.75), and 0.72 (95 % CI, 0.66–0.79), respectively. A likelihood ratio test was used to evaluate the effects of adding the term of stratified age for adverse events. Adding the age term for 0–19-, 20–39-, 60–79-, and ≥ 80-year-old groups, i.e., for all age groups, had statistically significant effects (*p* < 0.0001). The estimated values for the 0–19- and 20–39-year-old groups in the logistic regression exceeded 0 (estimated beta [β_3_] for 0–19-year-old group was 0.284, and that for the 20–39-year-old group was 0.579). Furthermore, we calculated the adjusted RORs for allopurinol, loxoprofen, acetaminophen, carbamazepine, and lamotrigine using the subset analysis. When considering the subset data for loxoprofen, the adjusted RORs for loxoprofen in patients in the 0–19-, 20–39-, 60–79-, and ≥ 80-year-old groups were 1.54 (95 % CI, 0.96–2.39), 1.89 (95 % CI, 1.49–2.39), 0.59 (95 % CI, 0.46–0.75), and 0.25 (95 % CI, 0.14–0.41), respectively. The likelihood ratio tests for the age terms of 20–39-, 60–79-, and ≥ 80-year-old groups had statistically significant effects (*p* < 0.0001). The adjusted RORs tended to be higher in the 20–39-year-old group. In contrast, the effect of the age term of the 0–19-year-old group was not statistically significant according to the likelihood ratio test (*p* = 0.0751). For acetaminophen, the adjusted RORs in patients in the 0–19-, 20–39-, 60–79-, and ≥ 80-year-old groups were 1.26 (95 % CI, 0.94–1.70), 1.31 (95 % CI, 0.95–1.79), 0.83 (95 % CI, 0.60–1.16), and 0.33 (95 % CI, 0.16–0.62), respectively. The likelihood ratio tests for the age term of the ≥ 80-year-old group showed statistically significant effects (*p* = 0.0004); however, the effects of the age terms of the 0–19-, 20–39-, and 60–79-year-old groups for acetaminophen were not statistically significant (*p* = 0.1292, *p* = 0.0972, and *p* = 0.2818, respectively). For carbamazepine, the adjusted RORs in patients in the 0–19-, 20–39-, 60–79-, and ≥ 80-year-old groups were 1.09 (95 % CI, 0.76–1.55), 1.43 (95 % CI, 1.06–1.95), 1.52 (95 % CI, 1.15–2.00), and 0.66 (95 % CI, 0.36–1.12), respectively. The effects of the age term of the 20–39- and 60–79-year-old group in the likelihood test (*p* = 0.0209, and *p* = 0.0027, respectively) in the subset data of carbamazepine were statistically significant, and the adjusted ROR was elevated in 20–39- and 60–79-year-old groups for carbamazepine. The effects of the age terms in the 20–39- and 60–79-year-old groups were statistically significant. For allopurinol, the effects of term in any age group were not statistically significant. For lamotrigine, the adjusted RORs were low in the 0–19- and 20–39-year-old groups.

**Table 2:** Adjusted reporting odds ratio of SJS/TEN stratified by age

	Estimated beta	Likelihood ratio test	Adjusted ROR (95 % CI)	
Total				
Reporting Year	−0.025	<0.0001^b^	0.98	(0.97-0.98)
Gender female	0.104	<0.0001^b^	1.11	(1.06-1.17)
Age				
≤ 19 years	0.284	<0.0001^b^	1.33	(1.21-1.45)
20-39 years	0.579	<0.0001^b^	1.78	(1.65-1.93)
40–59 years^a^				
60–79 years	−0.349	<0.0001^b^	0.71	(0.66-0.75)
≥ 80 years	−0.327	<0.0001^b^	0.72	(0.66-0.79)
Allopurinol				
Reporting Year	0.005	0.7756	1.00	(0.97-1.04)
Gender female	0.630	<0.0001^b^	1.88	(1.54-2.29)
Age				
≤ 19 years	−1.515	0.0713	0.22	(0.01-1.11)
20-39 years	0.165	0.4712	1.18	(0.75-1.82)
40–59 years^a^				
60–79 years	−0.123	0.3285	0.88	(0.69-1.13)
≥ 80 years	0.073	0.6324	1.08	(0.80-1.45)
Loxoprofen				
Reporting Year	−0.027	0.0706	0.97	(0.95-1.00)
Gender female	−0.150	0.1154	0.86	(0.71-1.04)
Age				
≤ 19 years	0.429	0.0751	1.54	(0.96-2.39)
20-39 years	0.635	<0.0001^b^	1.89	(1.49-2.39)
40–59 years^a^				
60–79 years	−0.535	<0.0001^b^	0.59	(0.46-0.75)
≥ 80 years	−1.399	<0.0001^b^	0.25	(0.14-0.41)
Acetaminophen				
Reporting Year	0.023	0.1744	1.24	(0.89-1.73)
Gender female	0.287	0.0082^b^	1.33	(1.08-1.65)
Age				
≤ 19 years	0.230	0.1292	1.26	(0.94-1.70)
20-39 years	0.266	0.0972	1.31	(0.95-1.79)
40–59 years^a^				
60–79 years	−0.184	0.2818	0.83	(0.60-1.16)
≥ 80 years	−1.103	0.0004^b^	0.33	(0.16-0.62)
Carbamazepine				
Reporting Year	0.005	0.7666	1.00	(0.97-1.04)
Gender female	0.187	0.0765	1.21	(0.98-1.48)
Age				
≤ 19 years	0.083	0.6486	1.09	(0.76-1.55)
20-39 years	0.360	0.0209^b^	1.43	(1.06-1.95)
40–59 years^a^				
60–79 years	0.416	0.0027^b^	1.52	(1.15-2.00)
≥80 years	−0.419	0.1293	0.66	(0.36-1.12)
Lamotrigine				
Reporting Year	0.036	0.3618	1.04	(0.96-1.12)
Gender female	0.158	0.1978	1.17	(0.92-1.49)
Age				
≤ 19 years	−0.992	<0.0001^b^	0.37	(0.22-0.60)
20-39 years	−0.418	0.0034^b^	0.66	(0.50-0.87)
40–59 years^a^				
60–79 years	−0.176	0.2672	0.84	(0.61-1.14)
≥ 80 years	0.162	0.5984	1.18	(0.63-2.09)

^a^As reference

^b^Statistically significant

For the time-to-onset analysis, the JADER database contained 2,575,326 combinations of drugs and adverse drug reactions (ADRs). After extracting the combinations having complete information for the date of starting treatment and the date of adverse event onset, 2,337,656 combinations were analyzed. A total of 21,502 events were identified for all drugs associated with SJS/TEN. From these, we analyzed the top five most frequently listed drugs, i.e., allopurinol, loxoprofen, acetaminophen, carbamazepine, and lamotrigine (Table 1). We also evaluated phenytoin, furosemide, angiotensin-converting enzyme (ACE) inhibitors, and corticosteroids. Lamotrigine, phenytoin and furosemide were evaluated in our previous report [9]. Furthermore, ACE inhibitors (i.e., imidapril hydrochloride, enalapril maleate, temocapril hydrochloride, lisinopril hydrate, perindopril erbumine, and alacepril) and corticosteroids (i.e., dexamethasone, dexamethasone sodium phosphate, dexamethasone acetate, triamcinolone acetonide, prednisolone, prednisolone acetate, betamethasone, betamethasone/d-chlorpheniramine maleate mixture, betamethasone valerate/gentamicin sulfate, methylprednisolone, and methylprednisolone sodium succinate), which were evaluated by Mockenhaupt et al., were summarized [6].

**Fig. 1:**
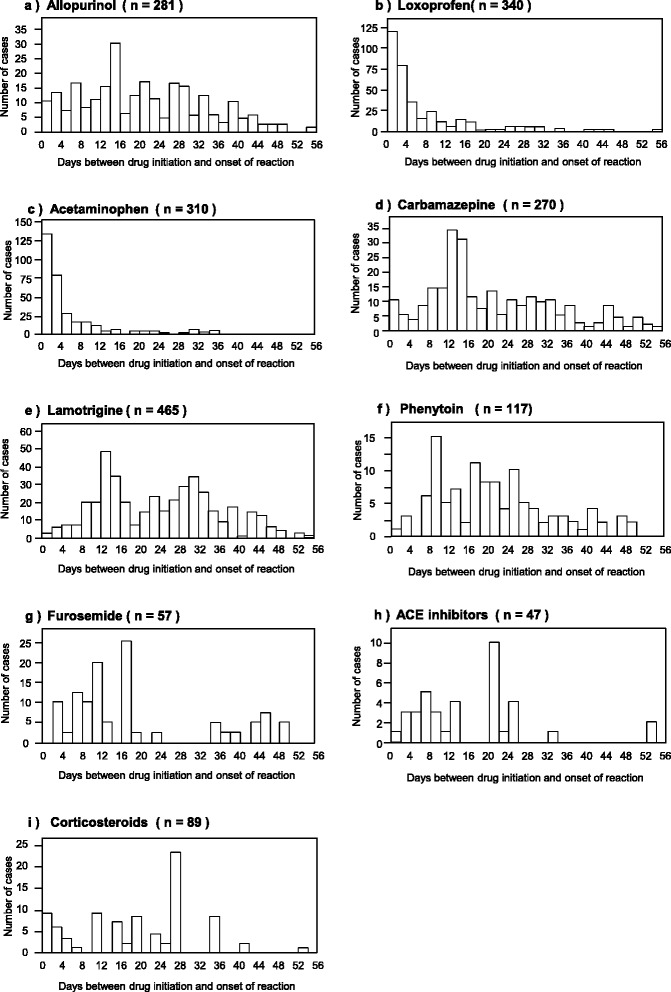
Histgram for time-to-onset profile of the SJS/TEN for the suspected drugs: (**a**) Allopurinol, (**b**) Loxoprofen, (**c**) Acetaminophen, (**d**) Carbamazepine, (**e**) Lamotrigine, (**f**) Phenytoin, (**g**) Furosemide, (**h**) ACE inhibitors, and (**i**) Corticosteroids

The results of the time-to-onset analysis are summarized in Table 3. The numbers of case reports for allopurinol, loxoprofen, acetaminophen, carbamazepine, lamotrigine, phenytoin, furosemide, ACE inhibitors, and corticosteroids were 281, 340, 310, 270, 465, 117, 57, 47, and 89, respectively. Figure 1 shows a histogram of the number of cases of SJS/TEN from days 0 to 56. The median durations (interquartile ranges) for SJS/TEN caused by allopurinol, loxoprofen, acetaminophen, carbamazepine, lamotrigine, phenytoin, furosemide, ACE inhibitors, and corticosteroids were 21 (12–35), 3 (1–8), 2 (1–5), 19 (12–33), 27 (14–37), 20 (11–29), 17 (9–45), 20 (6–33), and 19 (11–26) days, respectively. More than half of the reports of SJS/TEN onset following administration of loxoprofen and acetaminophen were recorded within 4 days of the initiation of treatment (Fig. 1). Furthermore, the scale parameter α values of loxoprofen and acetaminophen were 9.44 and 6.17, respectively. The upper 95 % CIs of shape parameter β values for the drugs were all less than 1, with the exceptions of those for carbamazepine, ACE inhibitors and corticosteroids.

**Table 3:** Quartile and parameter of Weibull distribution and failure pattern for each drugs

Drugs	Case reports	Median (day)	Lower quartile (day)	Upper quartile (day)	Minimum (day)	Maximum (day)	Scale parameter			Shape parameter		
							α	95 % CI		β	95 % CI	
Allopurinol	281	21	12	35	0	1689	45.66	37.23	55.85	0.62	0.58	0.67
Loxoprofen	340	3	1	8	0	708	9.44	7.74	11.49	0.64	0.59	0.69
Acetaminophen	310	2	1	5	0	191	6.17	5.17	7.35	0.74	0.68	0.81
Carbamazepine	270	19	12	33	0	451	31.20	27.52	35.32	1.02	0.94	1.10
Lamotrigine	465	27	14	37	0	962	46.66	40.66	53.49	0.71	0.67	0.75
Phenytoin	117	20	11	29	1	1109	30.46	23.76	38.92	0.79	0.70	0.88
Furosemide	57	17	9	45	2	434	34.14	23.32	49.30	0.75	0.62	0.91
ACE inhibitors^a^	47	20	6	33	0	227	38.02	25.62	55.48	0.81	0.65	1.00
Corticosteroids^b^	89	19	11	26	0	206	25.01	20.56	30.30	1.17	1.00	1.35

^a^It containd 6 drugs; imidapril hydrochloride, enalapril maleate, temocapril hydrochloride, lisinopril hydrate, perindopril erbumine, and alacepril

^b^It containd 11 drugs; dexamethasone, dexamethasone sodium phosphate, dexamethasone acetate, triamcinolone acetonide, prednisolone, prednisolone acetate, betamethasone, betamethasone/d-chlorpheniramine maleate mixture, betamethasone valerate/gentamicin sulfate, methylprednisolone, and methylprednisolone sodium succinate

## Discussion

In this study, we evaluated the relationship between aging and SJS/TEN using data from the JADER database. We also applied time-to-onset analysis using WSP tests. Our results provide important insights into the time-to-onset for SJS/TEN using available data.

In the JADER database, the top five most frequently administered drugs associated with SJS/TEN were allopurinol, loxoprofen, acetaminophen, carbamazepine, and lamotrigine. These drugs are known as causal drugs for SJS/TEN and are described in the Manual For Handling Disorders Due to Adverse Drug Reactions published by the Ministry of Health, Labour, and Welfare of Japan (“Koseirodosho” in Japanese); therefore, we believe that our results were reasonable. In contrast, in the FAERS database, the top five most frequently administered drugs associated with SJS/TEN were valdecoxib, lamotrigine, phenytoin, acetaminophen, and furosemide [9]. Because more than 70 % of reported cases from the U. S. were included in the FAERS database, the differences in prescriptions between Japan and the U. S. may explain these differences.

To the best of our knowledge, few studies have examined SJS/TEN in the context of age-group stratification. In this study, we examined the association between SJS/TEN and drugs using age-stratified data obtained from the JADER database. In a previous report, we described the adjusted RORs, stratified by age, for the FAERS database as follows: ≤17 years (2.87 [95 % CI, 2.67–3.07]), 18–39 years (1.46 [95 % CI, 1.37–1.55]), 40–59 years (1.00 [as a reference]), 60–79 years (0.99 [95 % CI, 0.93–1.04]), and ≥80-years (1.06 [95 % CI, 0.97–1.16]) [9]. In the FAERS database, the adjusted RORs in the youngest group (0–17 years) had the highest values of all data and subset data for all drugs (valdecoxib, lamotrigine, phenytoin, acetaminophen, and furosemide).

On the other hand, in the JADER database, the signal in the 0–19- and 20–39-year-old groups (1.33 [95 % CI, 1.21–1.45] and 1.78 [95 % CI, 1.65–1.93], respectively) was detected for the whole dataset (Table 2). The effects of the term for stratified age were statistically significant in all age groups. The adjusted RORs in the 0–19- and 20–39-year-old groups were higher than those in the 60–79- and ≥ 80-year-old groups. The ranges of the 95 % CI for the 0–19- and 20–39-year-old-groups were not overlapped with those for the 60–79- and ≥ 80-year-old-groups. SJS/TEN is thought to be an immune-mediated disease, and immune function decreases with age owing to the reduced function of T lymphocytes [21]. Consistent with these data and the results from our previous reports with the FAERS database [9], one plausible reason for this result may be differences in sensitivity to medication in younger versus older patients.

In addition to data mining approaches, data subsetting is useful for the evaluation of adverse event association in disproportionality analysis [22]. An intraclass ROR of the subset can be calculated after subsetting from the whole database [23]. The subset may be considered the same therapeutic class, which consists of a population of patients that presumably share a set of common risk factors and diseases. This strategy helps to mitigate confounding and bias [24]. Therefore, in this study, we calculated the adjusted ROR of the top five most frequently administered drugs associated with SJS/TEN using the subset analysis method. In the case of loxoprofen, the likelihood ratio tests for the age terms of the 20–39-, 60–79-, and ≥ 80-year-old groups had statistically significant effects. The adjusted RORs in the ≥ 80-year-old group for loxoprofen, acetaminophen, and carbamazepine were lowest among the stratified age groups. In our study, the age group having the highest adjusted ROR for each drug varied according to the drug. Genetic differences in the human Leukocyte antigen (HLA) have been reported as important factors contributing to the onset of SJS/TEN or hypersensitivity to drugs [10, 25]. Further studies are needed to determine the mechanisms through which these genetic factors explain our findings. Accordingly, to evaluate the true risk of medications, HLA genotyping should be evaluated in patients with varying ethnic backgrounds in well-organized epidemiological studies.

For acetaminophen, the adjusted RORs in patients in the 0–19- and 20–39-year-old groups were higher than those in the 60–79- and ≥ 80-year-old groups, although these differences were not significant (the range of the 95 % CI for the 60–79-year-old group was overlapped with those for the 0–19- and 20–39-year-old groups; Table 2). This may be explained by the potential inclusion of viral infection-induced SJS in data from the JADER database, which were obtained using the terms “SJS,” “oculomucocutaneous syndrome,” and “TEN.” SJS can be induced by mycoplasma infection, which is commonly observed in younger patients, and the treatment strategy for this condition would include antibiotics and/or acetaminophen. The co-administration ratio with antibiotics (63 antibiotics: cefcapene pivoxil hydrochloride hydrate, clarithromycin, cefdinir, etc.) was 44.9 % (864/1925) in the subset for acetaminophen; this was the highest value among the top five most frequently used drugs (allopurinol: 9.8 % [258/2642], loxoprofen: 28.7 % [1352/4714], carbamazepine: 6.0 % [272/4517], lamotrigine: 3.2 % [77/2375]). Therefore, the high incidence of SJS/TEN in young patients may be explained by acetaminophen treatment.

In the EuroSCAR-study, Mockenhaupt et al. examined the time course of SJS/TEN onset [6]. From their results of the time latency between the beginning of drug use and the onset of SJS/TEN, the time-to-onset duration of acetaminophen was less than 4 days. In our study, the median times-to-onset were 3 days for loxoprofen and 2 days for acetaminophen; these durations were relatively short in comparison with those of other drugs. Because the scale parameter α values of loxoprofen and acetaminophen were both less than 10, these small values indicate that there was a narrow data distribution. In the EuroSCAR Study, the median times-to-onset (interquartile ranges) of allopurinol, carbamazepine, phenytoin, and phenobarbital were 20 (14–32), 15 (12–20), 24 (16–33), and 17 (9–40) days, respectively. Our data for these allopurinol, carbamazepine, and phenytoin were similar to those in this previous study. Mockenhaupt et al. categorized the relationship between SJS/TEN and their examined drugs into three groups as follows: highly associated drugs (carbamazepine and nevirapine), nonassociated drugs (ACE inhibitors and valproic acid), and drugs with doubtful association (acetaminophen and corticosteroids) [6]. In our study, acetaminophen was one of the top five drugs associated with SJS/TEN. Therefore, patients receiving loxoprofen and acetaminophen treatment should be closely monitored for symptoms of SJS/TEN within the first 2–3 days of treatment in the Japanese population.

The time-to-onset analysis with the WSP method allows detection of potential adverse events without requiring a control population. In our study, we examined the time-to-onset of SJS/TEN using the WSP test; the results of the shape parameter β of the WSP indicated that the risk of SJS/TEN after administration of allopurinol, loxoprofen, acetaminophen, lamotrigine, phenytoin, and furosemide was decreased over time. In contrast, the risk of SJS/TEN with carbamazepine, ACE inhibitors, and corticosteroids remained almost constant. For corticosteroids, Mockenhaupt et al. speculated that the timing of corticosteroid exposure was caused by accumulation of cases during the same period as for associated drugs [6]. Notably, less than 100 case reports involved furosemide, ACE inhibitors, and corticosteroids; therefore, interpretation of data from such small sample sizes should be performed cautiously.

There are several limitations to this study, and the results obtained from SRSs, such as the JADER database, should be interpreted with caution. SRS is passive reporting system and is therefore subjected to many biases, such as under-reporting, over-reporting, and confounders by comorbidities. Most notably, there is a lack of comparison groups and missing data on patient characteristics [16, 26]. Because of these limitations, the crude RORs without logistic regression analysis do not indicate the risk of adverse event occurrence in absolute terms and can only offer a rough indication of signal strength [16]. In this study, we partially refined the results with a dedicated correction to detect possible confounders present in the database, using logistic regression and subset analysis. Despite this limitation, we believe it may be acceptable to compare the adjusted RORs of a particular adverse event derived from stratified analysis within a particular context. We hope that these data may enhance the information available to clinicians and improve the management of SJS/TEN.

## Conclusions

In summary, this study was the first to evaluate the association between aging and SJS/TEN using the JADER database and to apply the time-to-onset analysis technique to SJS/TEN using the SRS. We demonstrated that the adjusted RORs in patients 39 years old or younger were relatively high for the whole dataset and were particularly high in patients who were administrated antipyretic analgesics, such as loxoprofen or acetaminophen. More than half of the cases of SJS/TEN onset following administration of loxoprofen and acetaminophen were observed within 4 days. The median times-to-onset were 3 days for loxoprofen and 2 days for acetaminophen. Thus, patients receiving loxoprofen and acetaminophen treatment should be closely monitored for symptoms of SJS/TEN within the first 2–3 days of treatment in the Japanese population. Our results are useful for improving the management of SJS/TEN. Further research is needed in order to determine the specific associations between drugs and SJS/TEN.

## Abbreviations

ACE, angiotensin-converting enzyme; ADRs, adverse drug reactions; CI, confidence intervals; EuroSCAR Study, the European SCAR Study; FAERS, The United States Food and Drug Administration adverse event reporting system; FDA, Food and Drug Administration; HLA, the human Leukocyte antigen; JADER, the Japanese Adverse Drug Event Report; MedDRA, the medical dictionary for regulatory activities; PMDA, the Pharmaceuticals and Medical Devices Agency; PT, preferred terms; ROR, reporting odds ratio; SJS, Stevens-Johnson syndrome; SRSs, spontaneous reporting systems; TEN, toxic epidermal necrolysis; U. S., United States; WSP, the Weibull shape parameter
